# Enhanced-contrast optical readout in ultrafast broadband Raman quantum memories

**DOI:** 10.1038/s41598-018-31226-7

**Published:** 2018-09-13

**Authors:** A. M. Zheltikov

**Affiliations:** 1 Department of Physics and Astronomy, Texas A&M University, College Station, 77843 Texas USA; 2 Physics Department, International Laser Center, M.V. Lomonosov Moscow State University, Moscow, 119992 Russia; 3 Russian Quantum Center, Skolkovo, Moscow Region 143025 Russia; 4 Kazan Quantum Center, A.N. Tupolev Kazan National Research Technical University, Kazan, 420126 Russia

## Abstract

The signal-to-noise contrast of the optical readout in broadband Raman quantum memories is analyzed as a function of the pulse widths and phase properties of tailored optical field waveforms used to write in and read out broadband photon wave packets. Based on this analysis, we quantify the tradeoff between the readout contrast and the speed of such memories. Off-resonance coherent four-wave mixing is shown to provide a source of noise photons, lowering the readout contrast in broadband Raman quantum memories. This noise cannot be suppressed by phase matching, but can be radically reduced with a suitable polarization arrangement and proper field-waveform tailoring.

## Introduction

Optical quantum memories – systems capable of storing and maintaining quantum states of light, releasing them on demand – are of central importance for the emerging photonic quantum technologies^1,2^. Highly promising approaches to optical quantum memory have been demonstrated using ultracold atoms^3^, molecular gases^4^, atomic vapors^5^, rare-earth ion-doped crystals^6^, as well as phonons in room-temperature diamond^7–13^. Raman processes in ensembles of cold atoms have been shown to offer unique quantum-memory modalities, enabling polarization entanglement storage, as well as heralded single-photon entanglement of path and polarization storage^14^. Remarkably long coherence times have been demonstrated for a variety of quantum-memory systems operated at low temperatures^2^, including an astonishing six-hour coherence time in optically addressable nuclear spin systems in rare-earth ion-doped solids at 2 K^15^, opening new horizons in long-distance quantum communications.

As an alternative strategy, broadband quantum states of light, including broadband single-photon wave packets can be stored and retrieved on demand using vibrational and rotational modes of molecules^4,16^, as well as laser-driven phonons in diamond^7–13^. For this class of systems, coherence times are much shorter, typically ranging from a few picoseconds to several nanoseconds. However, unlike ultralong-coherence-time quantum memories, such shorter-lived memories can provide extremely broad, terahertz bandwidths and can function at room temperatures^7–13^. Moreover, such systems can operate at a much faster, femtosecond time scale, offering unique options for ultrahigh-speed quantum information technologies. Raman scattering provides an ideal write-in/read-out protocol for such broadband quantum memories^17–19^, allowing the quantum states of photons to be mapped onto molecular modes in gases or phonons^20^ in solids and enabling two-mode entanglement between light and relatively long-lived molecular or phonon modes in matter^21–23^.

Here, we analyze the tradeoff between the rate at which broadband Raman quantum memories can operate and the signal-to-noise contrast of the anti-Stokes readout from such memories. In crystalline solids, the readout contrast is strongly anisotropic, spanning many orders of magnitude for various polarization arrangements of the optical fields. Off-resonance coherent four-wave mixing (FWM) will be shown to provide a source of noise photons, lowering the readout contrast in broadband Raman quantum memories. We will demonstrate that this noise cannot be suppressed by phase matching, but can be reduced by many orders of magnitude with suitable polarization arrangements and proper field-waveform tailoring.

## Heisenberg-picture anti-Stokes readout analysis

**Figure 1:**
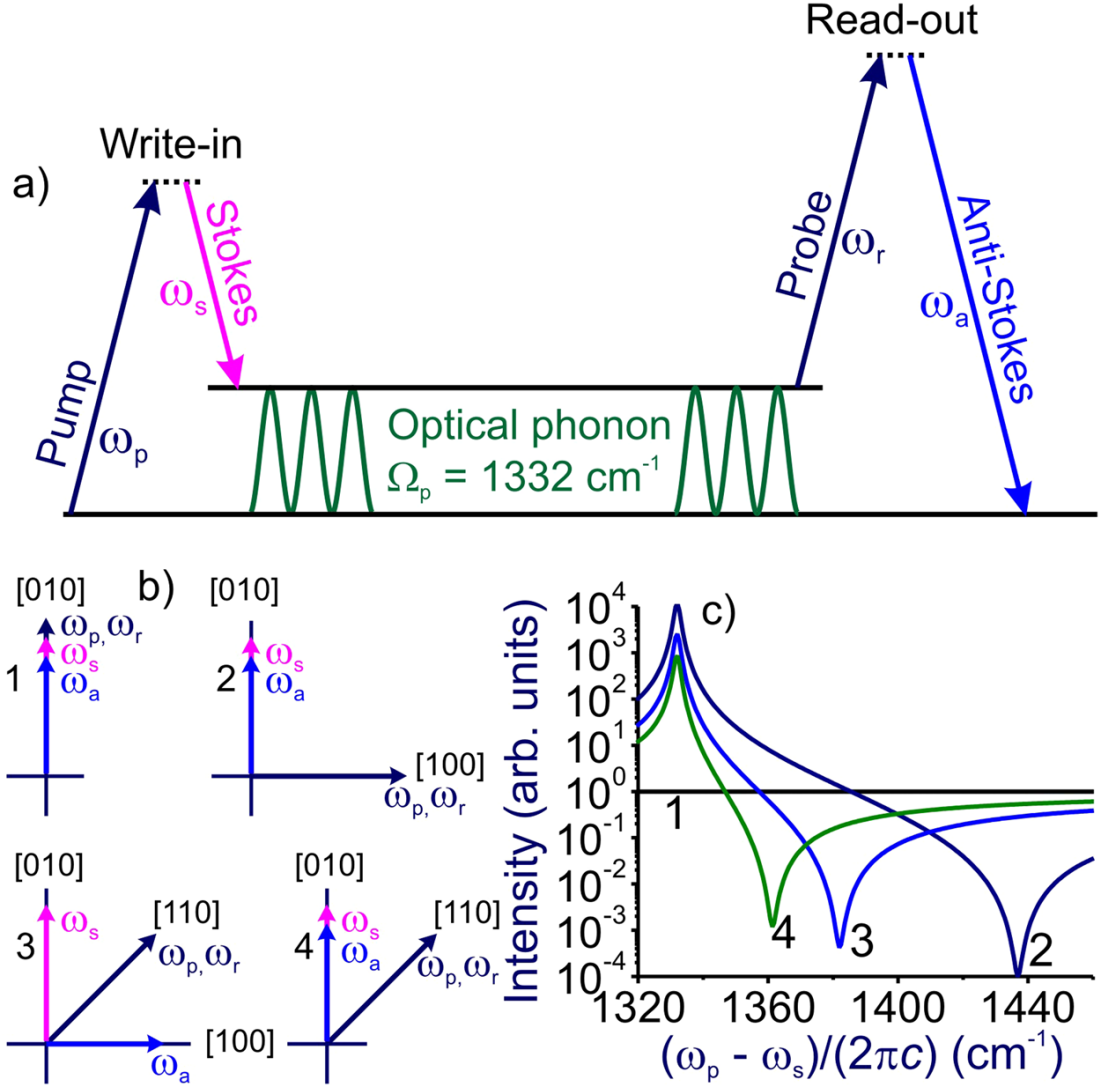
(**a**) Coherent anti-Stokes readout off the Raman quantum memory: (left) a frequency-tunable Stokes field maps the broadband pump wave packet into a memory based on coherently driven optical phonon, (right) the probe field applied with a variable delay time reads out the coherence driven by the pump and Stokes fields, giving rise to an anti-Stokes signal, which serves to report the state of the phonon memory. (**b**) Four polarization arrangements for the Raman quantum memory with anti-Stokes readout. Field polarization vectors are shown (arrows) against crystallographic directions. (**c**) The spectra of the anti-Stokes readout for different polarization arrangements as specified in panel (b) in the regime when the bandwidths of the pump, Stokes, and probe fields, Δ_p_, Δ_s_, and Δ_r_, are much smaller than the linewidth Γ of the Raman mode.

We consider optical readout signal generation in a Raman quantum memory scheme as a result of two-step coherent anti-Stokes Raman scattering (CARS) process involving write-in and read-out stages (Fig. 1a). CARS has been shown to provide a versatile spectroscopic protocol for the optical interrogation of long-lived coherent phonon modes in solids^24–27^, including the phonon modes in diamond, considered promising candidates for sold-state broadband memory^7–13^. At the first, write-in step, two fields with frequencies *ω*_p_ and *ω*_s_, referred to, in accordance with the standard CARS nomenclature^28,29^, as the pump and Stokes fields, drive a molecular or phonon mode with a central frequency Ω_R_ through a Raman-type resonance, *ω*_p_ − *ω*_s_ ≈ Ω_R_. At the second step, a probe field with a frequency *ω*_r_ reads out the Raman coherence, giving rise to an anti-Stokes signal at the central frequency *ω*_a_ = *ω*_p_ − *ω*_s_ + *ω*_r_, which serves to report the state of the phonon memory.

Field quantization is performed by replacing the slowly varying field envelopes *A*(*t*, *z*) by operators

1 $$\hat{A}(t,z)={(\frac{\hslash {\omega }_{0}}{{n}_{0}c})}^{1/2}\int \hat{a}(\omega )\exp [i({\beta }_{\omega }z-\omega t)]d\omega ,$$

where *ω*_0_ is the central frequency of the wave packet, *n*_0_ is the refractive index at the frequency *ω*_0_, *β*_*ω*_ is the propagation constant at the frequency *ω*, and $\hat{a}(\omega )$ is the field annihilation operator such that $[\hat{a}(\omega ),{\hat{a}}^{\dagger }(\omega ^{\prime} )]=\delta (\omega -\omega ^{\prime} )$.

The slowly varying field annihilation operator in the time domain is defined through the Fourier transform

2 $$\hat{a}(t,z)={(\frac{1}{2\pi })}^{1/2}\int \hat{a}(\omega ,z)\exp [i({\beta }_{\omega }z-\omega t)]d\omega ,$$

where $\hat{a}(\omega ,z)=\hat{a}(\omega )\exp (i{\beta }_{\omega }z)$, so that $[\hat{a}(\omega ,z),{\hat{a}}^{\dagger }(\omega ^{\prime} ,z)]=\delta (\omega -\omega ^{\prime} )$ and $[\hat{a}(t,z),{\hat{a}}^{\dagger }(t^{\prime} ,z)]=\delta (t-t^{\prime} )$.

In the second-quantization picture, the evolution of a quantum memory based on a Raman-active phonon driven by a classical pump field with a spectrum *A*_p_(*ω*) is described by an effective Hamiltonian^30^

3 $$\hat{H}^{\prime} =\hslash g{\hat{A}}_{{\rm{s}}}^{\dagger }{\hat{Q}}^{\dagger }+H.c.\,,$$

where ${\hat{Q}}^{\dagger }$ is the phonon creation operator, ${\hat{A}}_{{\rm{s}}}=\int {\rm{\Theta }}(\omega ){\hat{a}}_{{\rm{s}}}(\omega )d\omega$, ${\hat{a}}_{{\rm{s}}}(\omega )$ is the Stokes-field annihilation operator, ${\rm{\Theta }}(\omega )={A}_{p}(\omega ){[\int {|{A}_{p}(\omega ^{\prime} )|}^{2}d\omega ^{\prime} ]}^{-1/2}$, $g=\vartheta {[\int {|{A}_{p}(\omega ^{\prime} )|}^{2}d\omega ^{\prime} ]}^{1/2}$, and *ϑ* is the effective coupling constant.

The properties of such a Hamiltonians are discussed in an extensive literature on Raman quantum memory (see, e.g., refs^17,18^), as well as Raman protocols of quantum communication and quantum information processing^31^. The main focus of this work is on the limitations on the contrast of the optical readout in broadband Raman quantum memories due to the unwanted coherent FWM originating from off-resonance-driven electronic and two-photon excitations. The nonlinear signal produced through such FWM processes interferes with the anti-Stokes readout from the Raman coherence, dramatically lowering its contrast. Central to the analysis of this interference in classical-field models and experiments is the dispersion of the Raman-resonance and nonresonant parts of the pertinent nonlinear susceptibility^28,29,32^, that is, the frequency dependence of the Raman-resonant and nonresonant terms in the effective coupling constant. The goal of our analysis here is to extend these classical models to the Raman schemes operating with quantum states of the Stokes and anti-Stokes fields, such as Raman quantum memories. To this end, we continue treating the pump and readout (probe) fields classically and write the Hamiltonian as^33–35^

4 $$\hat{H}=\delta ({\hat{a}}_{a}^{\dagger }{\hat{a}}_{a}+{\hat{a}}_{s}^{\dagger }{\hat{a}}_{s})+\kappa ({\hat{a}}_{a}^{\dagger }{\hat{a}}_{s}^{\dagger }+{\hat{a}}_{a}{\hat{a}}_{s}),$$

where ${\hat{a}}_{a,s}=\hat{a}({\omega }_{a,s},z)$ ${\hat{a}}_{a,s}^{\dagger }={\hat{a}}^{\dagger }({\omega }_{a,s},z)$, $\delta ={\rm{\Delta }}\beta /2+\gamma ({P}_{p}+{P}_{r})/2$, *P*_p_ and *P*_r_ are the peak powers of the pump and probe fields, Δ*β* = *β*_a_ + *β*_s_ − *β*_p_ − *β*_r_, *β*_*j*_ = *β*(*ω*_*j*_), *j* = p, s, r, a, $\kappa =\varepsilon \gamma {A}_{p}{A}_{r}$, *ε* is the polarization-sensitive numerical factor, *P*_p_ and *P*_r_ are the complex amplitudes of the pump and probe fields, respectively, and *γ* is the nonlinear coefficient related to the pertinent third-order optical susceptibility.

For the full quantum analysis of the phonon modes behind the quantum memory, the evolution equations as dictated by the Hamiltonian (3) need to be included in the model. The Hamiltonian (4) is not intended for such an analysis. Instead, it provides an adequate framework for the description of the spectral interference of the anti-Stokes readout from the Raman coherence with off-resonance FWM, thus enabling a quantitative analysis of the readout contrast in Raman quantum memories.

With the Hamiltonian taken in the form of Eq. (4), the solution to the Heisenberg-picture evolution equations

5 $$d{\hat{a}}_{a,s}/dz=i[{\hat{a}}_{a,s},\hat{H}]$$

can be written in the input–output form as^33–35^

6 $${\hat{a}}_{a}(z)=\mu (z){\hat{a}}_{a}(0)+\nu (z){\hat{a}}_{s}^{\dagger }(0),$$

7 $${\hat{a}}_{s}^{\dagger }(z)={\mu }^{\ast }(z){\hat{a}}_{s}^{\dagger }(0)+{\nu }^{\ast }(z){\hat{a}}_{a}(0),$$

where $$\mu (z)=\,\cos (sz)+i(\delta /s)\sin (sz)$$, $$\nu (z)=i(\kappa /s)\sin (sz)$$, and $${s}^{2}={\delta }^{2}-{\kappa }^{2}$$.

For a rigorous analysis of ultrashort pulses, the full spatial derivatives *d*/*dz* in the evolution equations for ${\hat{a}}_{a,s}$ should be replaced by convective derivatives $\partial /\partial z+{u}_{a,s}^{-1}\partial /\partial t$, with *u*_s_ and *u*_a_ being the group velocities of the Stokes and anti-Stokes pulses. Here, however, we seek to isolate effects related to the pulse shape, phase, and polarization of optical fields. We therefore choose to work in the approximation where group walk-off effects are neglected – approximation that is broadly accepted even in ultrafast-CARS literature^36^.

We now use the Heisenberg picture to calculate the expectation value for the anti-Stokes field photon-number operator, $\langle {n}_{a}(z)\rangle =\langle \psi (z=0)|{a}_{a}^{\dagger }(z){a}_{a}(z)|\psi (z=0)\rangle$. With an input Stokes–anti-Stokes field state with no anti-Stokes photons at *z* = 0, $|\psi (z=0)\rangle =|s,0\rangle$, we find

8 $$\langle {n}_{a}(z)\rangle ={|\nu (z)|}^{2}(\langle {n}_{s0}\rangle +1),$$

where $$\langle {n}_{s0}\rangle =\langle {n}_{s}(z=0)\rangle $$.

Notably, with <*n*_*s*0_> = 0, corresponding to a vacuum input state, $|\psi (z=0)\rangle =|0,0\rangle$, Eq. (8) recovers the two-mode squeezed-state result of the input–output Heisenberg-picture analysis of FWM^33–35^, $\langle {n}_{a}(z)\rangle ={|\nu (z)|}^{2}$.

It is, however, the first, ∝<*n*_*s*0_> term of Eq. (8) that we are mainly concerned with here in our analysis of broadband Raman quantum memories. For |*κ*| ≪ |*δ*|, this part of the anti-Stokes readout can be written as

9 $$R(z)={|\nu (z)|}^{2}\langle {n}_{s0}\rangle ={|\gamma |}^{2}\frac{{\sin }^{2}({\rm{\Delta }}\beta z/2)}{{({\rm{\Delta }}\beta /2)}^{2}}{P}_{p}{P}_{r}\langle {n}_{s0}\rangle .$$

In the case of a classical Stokes field at the input, $\langle {n}_{a,s}\rangle \approx {(\hslash {\omega }_{a,s})}^{-1}{P}_{a,s}{\tau }_{a,s}$, where *P*_a,s_ are the peak powers of the anti-Stokes and Stokes pulses and *τ*_a,s_ are their respective pulse widths, the standard classical-field result is recovered:

10 $${P}_{a}(z)\propto {|\gamma |}^{2}\frac{{\sin }^{2}({\rm{\Delta }}\beta z/2)}{{({\rm{\Delta }}\beta /2)}^{2}}\frac{{\tau }_{s}}{{\tau }_{a}}\frac{{\omega }_{a}}{{\omega }_{s}}{P}_{p}{P}_{r}{P}_{s}.$$

## Off-resonance coherent four-wave mixing as a source of anisotropic photon noise

Apart from the complex, frequency-dependent Raman-resonant part, *γ*_*R*_(*ω*), the FWM nonlinear coefficient *γ* generally includes a purely real nonresonant term, *γ*_*nr*_, related to electron transitions^28,29,32,37^, and may include a distinct two-photon-resonant term^38,39^, *γ*_*tp*_, which is of special significance in solid semiconductors,

11 $$\gamma ={\gamma }_{R}(\omega )+{\gamma }_{nr}+{\gamma }_{tp}.$$

For cubic and isotropic materials, the spectrum of the Raman resonance can be approximated with a Lorentzian profile, leading to the following three-term approximation for |*γ*|^2^ near the Raman resonance ${\omega }_{p}-{\omega }_{s}\approx {{\rm{\Omega }}}_{R}$^38,39^:

12 $${|\gamma ({\omega }_{p}-{\omega }_{s})|}^{2}\propto {|1-i\frac{{\chi }_{2}}{{\chi }_{1}}+\frac{N{|{\alpha }_{R}|}^{2}}{4\hslash {\chi }_{1}}\frac{1}{{{\rm{\Omega }}}_{R}-({\omega }_{p}-{\omega }_{s})+i{\rm{\Gamma }}}|}^{2},$$

where *N* is the number density of Raman-resonant species, Γ is the linewidth of the Raman resonance, *α*_R_ is the local-field-corrected polarizability, *χ*_1_ and *χ*_2_ are the real and imaginary parts of the effective cubic susceptibility *χ*_*eff*_ = *χ*_1_ − i*χ*_2_, which can be expressed through the components of the electronic third-order susceptibility ${\chi }_{ijkl}^{(3)E}$ as ${\chi }_{eff}={d}_{1}{\chi }_{1111}^{(3)E}+{d}_{2}{\chi }_{1221}^{(3)E}+{d}_{3}{\chi }_{1122}^{(3)E}$. The coefficients *d*_1_, *d*_2_, and *d*_3_ depend on the polarization arrangement of optical fields, reflecting tensor properties of the electronic susceptibility ${\chi }_{ijkl}^{(3)E}$ and polarizability *α*_R_. For four representative arrangements of the pump, Stokes, probe, and anti-Stokes polarization vectors relative to the [100] direction of the diamond lattice sketched in Fig. 1, we find no Raman resonance for polarization geometry 1 (curve 1 in Fig. 1c), *d*_1_ = 0, *d*_2_ = 6, and *d*_3_ = 0 for geometry 2 (curve 2 in Fig. 1c), *d*_1_ = 0, *d*_2_ = 0, and *d*_3_ = 12 for geometry 3 (curve 3 in Fig. 1c), and *d*_1_ = 6, *d*_2_ = 6, and *d*_3_ = 0 for geometry 4 (curve 4 in Fig. 1c).

The anti-Stokes signal is thus a result of spectral interference of the photon fields generated through the CARS-type scattering of the probe field off the Raman coherence, encoding the information written by the pump and Stokes fields, and the nonresonant FWM background related to the *γ*_*nr*_ and *γ*_*tr*_ terms in Eq. (11). Both the useful anti-Stokes Raman memory readout signal and the FWM background build up as sin^2^(Δ*βz*/2) as functions of the propagation path. As a consequence, the nonresonant background in anti-Stokes photon counts cannot be suppressed by phase-matching adjustments.

## Polarization-sensitive anti-Stokes readout contrast enhancement

As can be seen from Eq. (12), the ratio $\eta =N{|{\alpha }_{R}|}^{2}/(4\hslash {\chi }_{1})$ [the factor that appears in front of the frequency denominator in the third term in Eq. (12)] provides a meaningful quantitative measure for the contrast of the anti-Stokes memory readout relative to the FWM nonresonant background. This ratio is highly sensitive to the polarizations of the optical fields used to write information into the Raman memory and to read out the state of this memory.

Analysis of the tensor properties of ${\chi }_{ijkl}^{(3)E}$ and polarizability *α*_R_ of diamond shows that this material is, in many respects, a highly promising medium for quantum memory. The cubic symmetry of the crystal lattice of diamond provides an ample parameter space for *η* contrast optimization. At the same time, the 1332-cm^−1^ zone-center Γ^(25+)^ (*F*_2*g*_) symmetry optical phonon in diamond, offers an advantageous combination of a broad, terahertz bandwidth and a sufficiently long coherence time in the range of a few picoseconds, allowing ultrafast Raman memory schemes to be implemented at room temperatures using femtosecond laser pulses^7–13^.

However, a great care needs to be exercised in choosing polarizations of the laser beam to provide the highest possible anti-Stokes-readout-to-FWM-background ratios. In particular, for the polarization arrangement where the pump field is polarized along the [100] direction of the diamond lattice, while the Stokes and probe fields are polarized along the [010] direction (polarization geometry 2 in Fig. 1b), we have *η* ≈ 110 cm^−1^ and |*χ*_2_/*χ*_1_| ≪ 1^38,39^, providing a high-contrast anti-Stokes readout against a virtually negligible FWM background (curve 2 in Fig. 1c).

Storing polarization qubits, however, requires two different polarization arrangements of optical fields. Building upon the high *η* ratios provided by the polarization arrangement 2 in Fig. 1b, it is tempting to use a scheme with all the laser fields polarized along the [010] direction (polarization geometry 1 in Fig. 1b) as the second polarization arrangement for polarization-qubit storage. However, the triply degenerate *F*_2*g*_-symmetry optical phonon in diamond (as well as in CaF_2_ and other homologous fluorides) has zero Raman-resonant cubic susceptibility ${\chi }_{1111}^{(3)R}$^38,39^. The useful anti-Stokes readout vanishes in this polarization arrangement (curve 1 in Fig. 1c). With this exception, diamond provides a vast parameter space for ultrafast broadband quantum memory with a high-contrast anti-Stokes readout (Fig. 1c).

## Broadband photon wave packets

In the case of ultrashort pump, Stokes, and probe field waveforms, ${E}_{j}(t,z)={A}_{j}(t,z)\exp [i({\beta }_{j}z-{\omega }_{j}t)]$, *j* = p, r, s, the integration over the broadband photon packets representing these waveforms in the frequency domain, with $|\psi (z=0)\rangle =|s,0\rangle$ and |Δ*βz*| ≪ 1, yields

13 $$\begin{array}{rcl}\langle {n}_{a}(\omega ,z)\rangle  & = & \langle \psi (z=0)|{a}_{a}^{\dagger }(\omega ,z){a}_{a}(\omega ,z)|\psi (z=0)\rangle \\  & = & \iint d{\omega ^{\prime} }_{s}d{\rm{\Omega }}^{\prime} {\gamma }^{\ast }({\rm{\Omega }}^{\prime} ){A}_{p}^{\ast }({\omega ^{\prime} }_{s}+{\rm{\Omega }}^{\prime} ){A}_{r}^{\ast }(\omega -{\rm{\Omega }}^{\prime} ){a}_{s}({\omega ^{\prime} }_{s})\\  &  & \iint d{\omega }_{s}d{\rm{\Omega }}\gamma ({\rm{\Omega }}){A}_{p}({\omega }_{s}+{\rm{\Omega }}){A}_{r}(\omega -{\rm{\Omega }}){a}_{s}^{\dagger }({\omega }_{s})\end{array}$$

With the quantization rule of Eqs (3 and 13) reduces to

14 $$\begin{array}{rcl}\langle {n}_{a}(\omega ,z)\rangle  & = & \int d{\rm{\Omega }}^{\prime} {\gamma }^{\ast }({\rm{\Omega }}^{\prime} ){A}_{r}^{\ast }(\omega -{\rm{\Omega }}^{\prime} )\int d{\rm{\Omega }}\gamma ({\rm{\Omega }}){A}_{r}(\omega -{\rm{\Omega }})\\  &  & \int d{\omega }_{s}{A}_{p}^{\ast }({\omega }_{s}+{\rm{\Omega }}^{\prime} ){A}_{p}({\omega }_{s}+{\rm{\Omega }})(\langle {n}_{s0}({\omega }_{s})1\rangle +1)\end{array}$$

The anti-Stokes readout of the Raman quantum memory is thus given by

15 $$\begin{array}{rcl}R(\omega ,z) & = & \int d{\rm{\Omega }}^{\prime} {\gamma }^{\ast }({\rm{\Omega }}^{\prime} ){A}_{r}^{\ast }(\omega -{\rm{\Omega }}^{\prime} )\int d{\rm{\Omega }}\gamma ({\rm{\Omega }}){A}_{r}(\omega -{\rm{\Omega }})\\  &  & \int d{\omega }_{s}{A}_{p}^{\ast }({\omega }_{s}+{\rm{\Omega }}^{\prime} ){A}_{p}({\omega }_{s}+{\rm{\Omega }})\langle {n}_{s0}({\omega }_{s})\rangle \end{array}$$

As the first important limiting regime, we consider the case when the bandwidths of the laser pulses Δ_p_ and Δ_s_ are much smaller than the linewidth Γ of the Raman resonance,

16 $${{\rm{\Delta }}}_{{\rm{p}}},{{\rm{\Delta }}}_{{\rm{s}}}\ll {\rm{\Gamma }},$$

so that $$\int d{\omega }_{s}{A}_{p}^{\ast }({\omega }_{s}+{\rm{\Omega }}^{\prime} ){A}_{p}({\omega }_{s}+{\rm{\Omega }})\langle {n}_{s0}({\omega }_{s})\rangle ={P}_{p}\langle {n}_{s0}\rangle \delta ({\rm{\Omega }}-{{\rm{\Omega }}}_{R})\delta ({\rm{\Omega }}^{\prime} -{{\rm{\Omega }}}_{R})$$.

With the nonlinear coefficient defined by Eq. (12) with |*χ*_2_/*χ*_1_| ≪ 1, as in the case of diamond, Eq. (15) is reduced in this regime to

17 $$R(\omega ,z)\approx [1+{(\eta /{\rm{\Gamma }})}^{2}]{P}_{p}{|{A}_{r}(\omega -{{\rm{\Omega }}}_{R})|}^{2}\langle {n}_{s0}\rangle .$$

This result recovers Eq. (9) with |*γ*| as defined by Eq. (12), |Δ*βz*| ≪ 1, and ${\omega }_{p}-{\omega }_{s}\approx {{\rm{\Omega }}}_{R}$.

**Figure 2:**
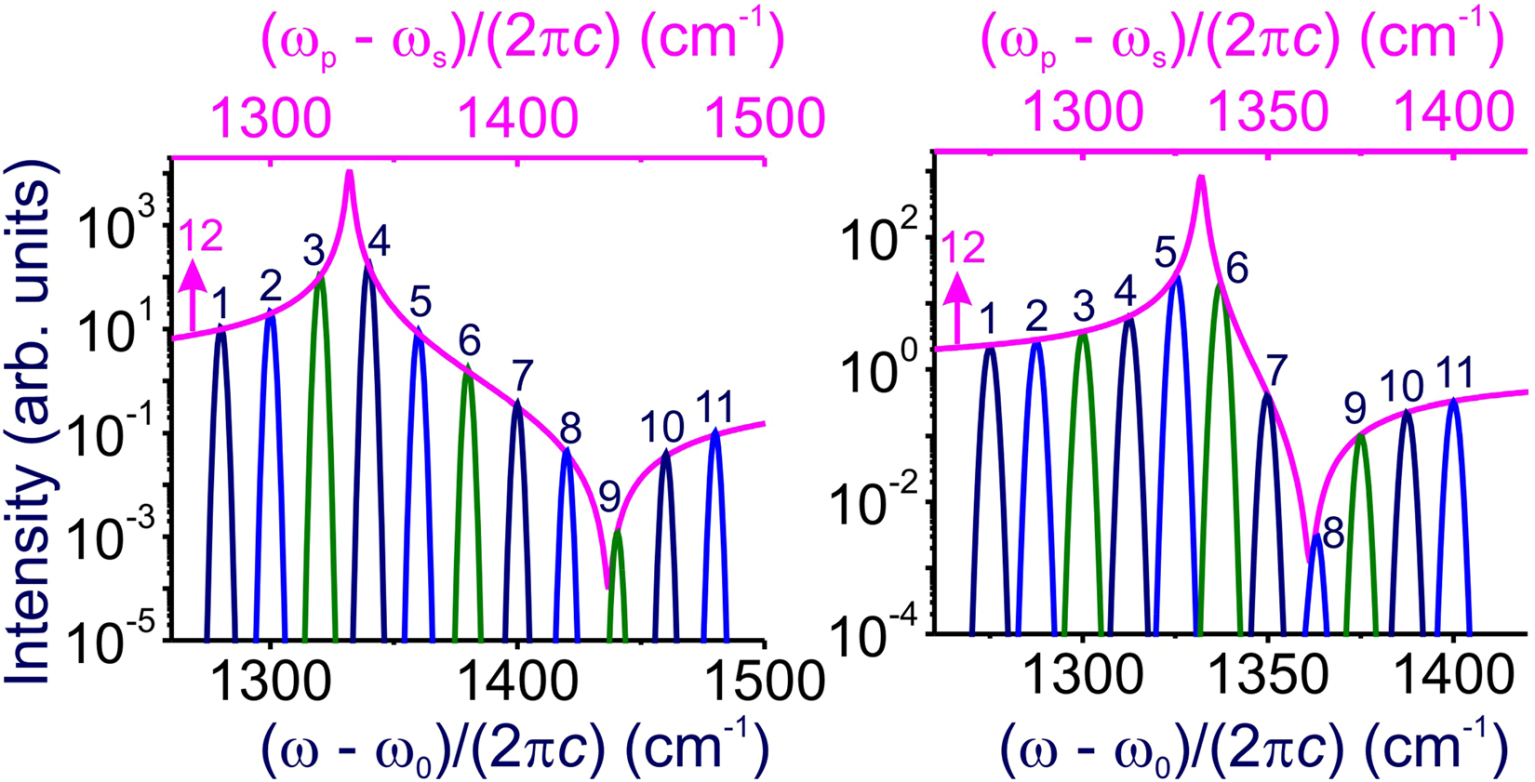
Spectra of the anti-Stokes readout from the Ω_R_ ≈ 1332 cm^−1^ zone-center Γ^(25+)^ (*F*_2*g*_) symmetry optical phonon in diamond driven and probed in the polarization geometry 2 (**a**) and 4 (**b**). The pulse widths of the pump and Stokes pulses are 10 ps. The central wavelength of the pump pulse is 800 nm. The pump–Stokes frequency offset (*ω*_*p*_ − *ω*_*s*_)/(2*πc*) is (**a**) 1280 cm^−1^ (1), 1300 cm^−1^ (2), 1320 cm^−1^ (3), 1340 cm^−1^ (4), 1360 cm^−1^ (5), 1380 cm^−1^ (6), 1400 cm^−1^ (7), 1420 cm^−1^ (8), 1440 cm^−1^ (9), 1460 cm^−1^ (10), and 1480 cm^−1^ (11); (**b**) 1275 cm^−1^ (1), 1283 cm^−1^ (2), 1300 cm^−1^ (3), 1313 cm^−1^ (4), 1325 cm^−1^ (5), 1338 cm^−1^ (6), 1350 cm^−1^ (7), 1363 cm^−1^ (8), 1375 cm^−1^ (9), 1388 cm^−1^ (10), and 1400 cm^−1^ (11). Curve 12 shows the spectrum of the anti-Stokes readout in the regime when the bandwidths of the pump, Stokes, and probe fields, Δ_p_, Δ_s_, and Δ_r_, are much smaller than the linewidth Γ of the Raman mode.

The highest contrast of the anti-Stokes signal relative to the FWM background achieved in this regime is *σ*_0_ ≈ |*η*|^2^/Γ^2^. For the 1332-cm^−1^ zone-center Γ^(25+)^ (*F*_2*g*_) symmetry optical phonon in diamond driven and probed in polarization arrangements 2, as shown in Fig. 1b, *η*_2_ ~ 110 cm^−1^ and Γ ~ 1 cm^−1^ ^38,39^. The highest contrast of the anti-Stokes signal in this scheme is *σ*_0_ ~ 10^4^ (Fig. 2a). For the polarization arrangement 4 (Fig. 1b), the contrast of the anti-Stokes readout is seen to be about 13 times lower (cf. Fig. 2a,b). This correlates well with the polarization properties of the Raman response of the 1332-cm^−1^ phonon mode. Indeed, for the polarization arrangements 4, *η*_4_ ~ 30 cm^−1^ ^38,39^. Correspondingly, the ratio of the peak values of the anti-Stokes signal in polarization geometries 2 and 4 in Fig. 1c is ~13, leading to a 13-fold difference in the highest contrast of the anti-Stokes readout for the polarization arrangements 2 and 4 in Fig. 2a,b.

As the pump–Stokes frequency difference *ω*_*p*_ − *w*_*s*_ is scanned near and through the Raman resonance ${\omega }_{p}-{\omega }_{s}={{\rm{\Omega }}}_{R}$, the maximum of the anti-Stokes readout in the Δ_p_, Δ_s_ ≪ Γ regime closely follows the spectral profile of the Raman mode (pink curve 12 in Fig. 2a,b). In the context of ultrafast broadband quantum memory, however, such a high contrast is achieved at the expense of the operation speed. Indeed, in the case of a diamond-based quantum memory, the condition Δ_p_, Δ_s_ ≪ Γ is satisfied for laser pulse widths longer than or on the order of 10 ps, which limits the memory speed to the 1–10-GHz rate level.

In the opposite limiting case, when the bandwidths of the laser pulses Δ_p_, Δ_s_, and Δ_r_ are much broader than the linewidth Γ, Eq. (15) with |*γ*| as defined by Eq. (12) with |*χ*_2_/*χ*_1_| ≪ 1 leads to

18 $$R(\omega ,z)\approx [1+{\eta }^{2}/({{\rm{\Delta }}}_{p}^{2}+{{\rm{\Delta }}}_{s}^{2})]{|{A}_{p}({\omega }_{p})|}^{2}{|{A}_{r}({\omega }_{r})|}^{2}\langle {n}_{s0}\rangle .$$

**Figure 3:**
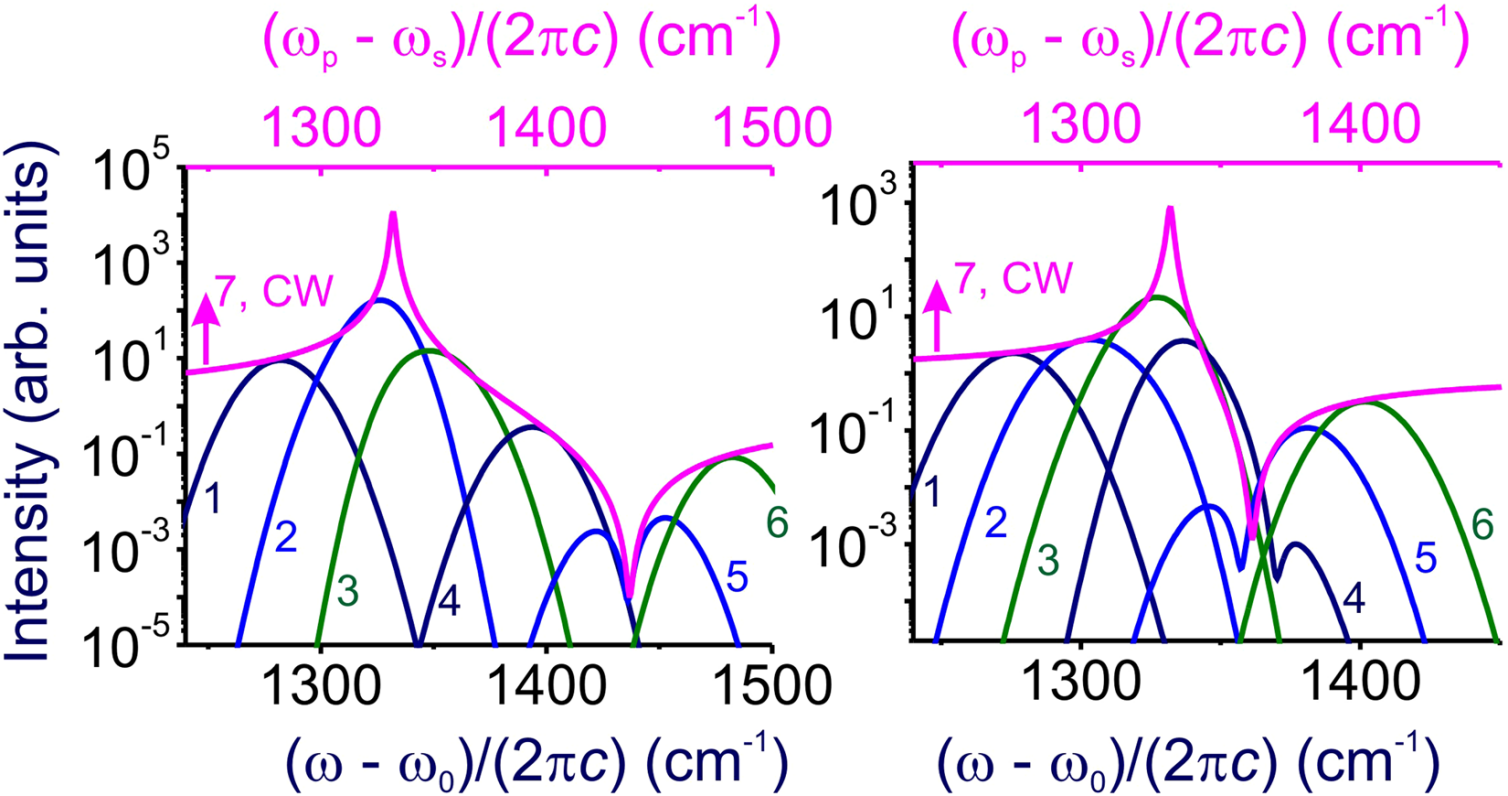
Spectra of the anti-Stokes readout from the Ω_R_ ≈1332 cm^−1^ zone-center Γ^(25+)^ (*F*_2*g*_) symmetry optical phonon in diamond driven and probed in the polarization geometry 2 (**a**) and 4 (**b**). The pulse widths of the pump and Stokes pulses are 1 ps. The central wavelength of the pump pulse is 800 nm. The pump–Stokes frequency offset (*ω*_*p*_ − *ω*_*s*_)/(2*πc*) is (**a**) 1280 cm^−1^ (1), 1320 cm^−1^ (2), 1360 cm^−1^ (3), 1400 cm^−1^ (4), 1440 cm^−1^ (5), and 1480 cm^−1^ (6); (**b**) 1275 cm^−1^ (1), 1300 cm^−1^ (2), 1325 cm^−1^ (3), 1350 cm^−1^ (4), 1375 cm^−1^ (5), and 1400 cm^−1^ (6). Curve 7 shows the spectrum of the anti-Stokes readout in the regime when the bandwidths of the pump, Stokes, and probe fields, Δ_p_, Δ_s_, and Δ_r_, are much smaller than the linewidth Γ of the Raman mode.

**Figure 4:**
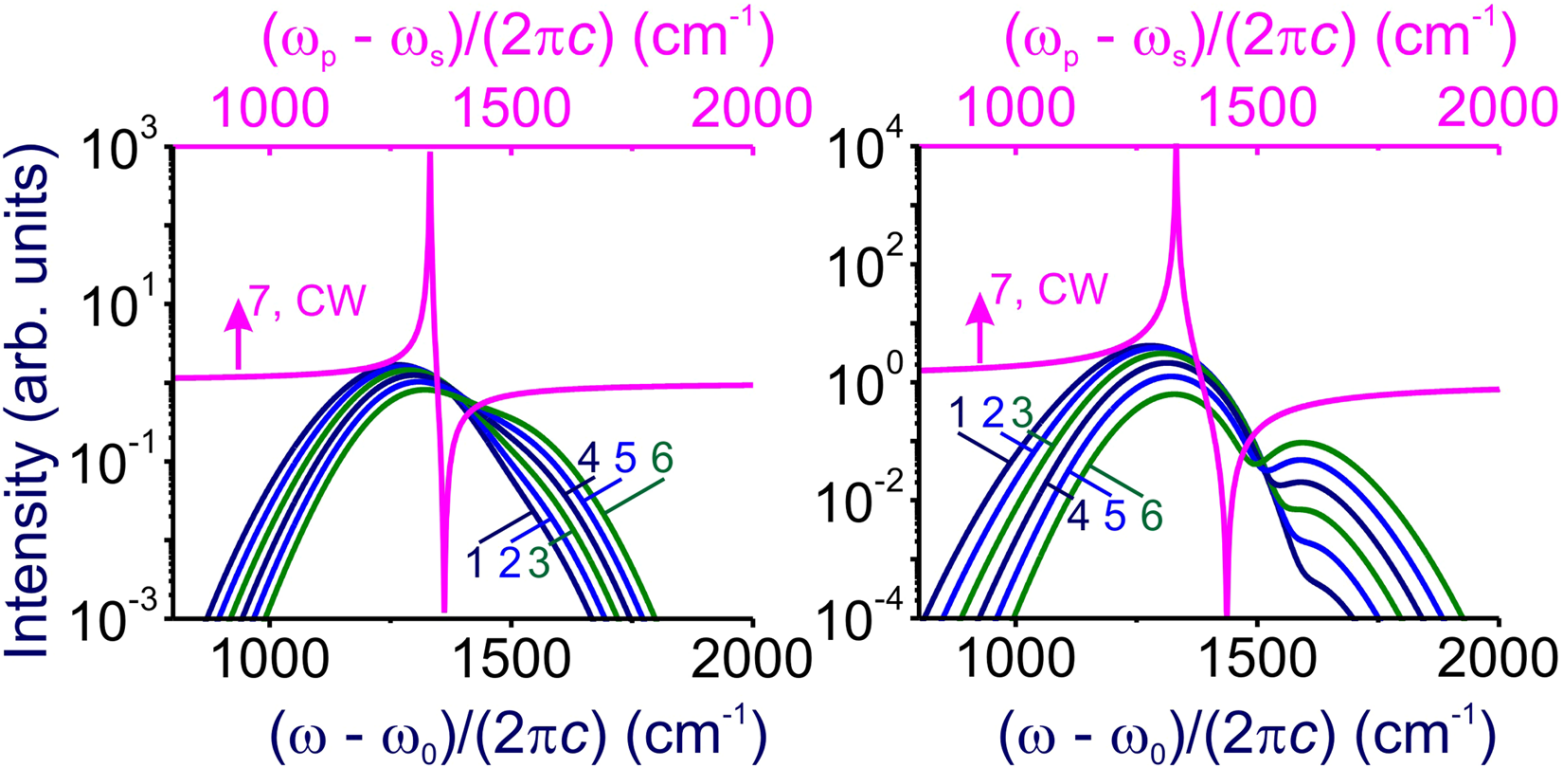
Spectra of the anti-Stokes readout from the Ω_R_ ≈1332 cm^−1^ zone-center Γ^(25+)^ (*F*_2*g*_) symmetry optical phonon in diamond driven and probed in the polarization geometry 2 (**a**) and 4 (**b**). The pulse widths of the pump and Stokes pulses are 100 fs. The central wavelength of the pump pulse is 800 nm. The pump–Stokes frequency offset (*ω*_*p*_ − *ω*_*s*_)/(2*πc*) is (**a**) 1280 cm^−1^ (1), 1320 cm^−1^ (2), 1360 cm^−1^ (3), 1400 cm^−1^ (4), 1440 cm^−1^ (5), and 1480 cm^−1^ (6); (**b**) 1275 cm^−1^ (1), 1300 cm^−1^ (2), 1325 cm^−1^ (3), 1350 cm^−1^ (4), 1375 cm^−1^ (5), and 1400 cm^−1^ (6). Curve 7 shows the spectrum of the anti-Stokes readout in the regime when the bandwidths of the pump, Stokes, and probe fields, Δ_p_, Δ_s_, and Δ_r_, are much smaller than the linewidth Γ of the Raman mode.

In this case, the contrast of the anti-Stokes readout signal relative to the FWM background is a factor of $({{\rm{\Delta }}}_{p}^{2}+{{\rm{\Delta }}}_{s}^{2})/{{\rm{\Gamma }}}^{2}$ lower (Figs 3 and 4) than its nominally highest value, *σ*_0_ ≈ |*η*|^2^/Γ^2^. Specifically, with ~100-fs laser pulses, 10-THz operation rates can be achieved for solid-state quantum memory based on the 1332-cm^−1^ optical phonon in diamond. However, with Γ ~ 1 cm^−1^, we find $({{\rm{\Delta }}}_{p}^{2}+{{\rm{\Delta }}}_{s}^{2})/{{\rm{\Gamma }}}^{2} \sim {10}^{4}$ for such pulse width, indicating much lower readout contrasts (Fig. 4).

Similar to their classical-field analogues, Eqs (12, 13, 15 and 17) suggest that the contrast of the anti-Stokes readout can be enhanced through a proper field-waveform tailoring. Indeed, the maximum level of the nonresonant FWM signal is achieved, as can be seen from these expressions, when the pump, Stokes, and probe pulses are all transform-limited. Resonant excitation of Raman-active phonon, on the other hand, does not necessarily require transform-limited pump and Stokes fields. Phase modulated pump and Stokes pulses can still provide a resonant excitation of a phonon mode with a central frequency Ω_R_ as long as their spectral phase functions Φ_p_(*ω*) and Φ_s_(*ω*) satisfy the condition^36,40^ Φ_p_(*ω*) = Φ_s_(*ω* − Ω_R_). In CARS microscopy, this condition, as elegantly shown in refs^36,40^, can be satisfied by applying frequency-shifted step functions to the spectral phases of the pump and Stokes fields. The Φ_p_(*ω*) = Φ_s_(*ω* − Ω_R_) recipe can be extended to Raman quantum information processing and storage protocols operating with quantum states of light with a well-defined phase. In the Raman scheme considered in this work, this implies a well-defined phase of the Stokes field, Φ_s_(*ω*). As is also readily seen from Eqs (13, 15 and 17), uncertainty in the phases of optical fields translate into fluctuations of the anti-Stokes readout. The quantum-field treatment of coherent anti-Stokes Raman scattering provided in the previous sections thus remains meaningful beyond Raman memory schemes, providing a closed formalism for a quantitative analysis of the anti-Stokes readout in coherent Raman scattering of quantum optical fields.

## Anti-Stokes readout with a time-delayed probe

As a powerful resource for a practical implementation of quantum memory, the probe (reading) pulse in broadband Raman memory can be applied with a variable delay time *τ*_d_ relative to the pump–Stokes write-in pulse dyad (Fig. 1a). To appreciate the advantages of this modality, it is instructive to transform the nonlinear susceptibility ${\chi }^{(3)}({\omega }_{a};{\omega }_{p},-{\omega }_{s},{\omega }_{r})$, controlling, via Eq. (12), the nonlinear coefficient in the *ω*_a_ = *ω*_p_ − *ω*_s_ + *ω*_r_ coherent Raman memory, to the time domain:

19 $${\chi }^{(3)}({\omega }_{a};{\omega }_{p},-{\omega }_{s},{\omega }_{r})=\iiint {\chi }^{(3)}({t}_{1},{t}_{2},{t}_{3})\exp [i({\omega }_{p}{t}_{1}-{\omega }_{s}{t}_{2}+{\omega }_{r}{t}_{3})]d{t}_{1}d{t}_{2}d{t}_{3}.$$

We isolate the Raman-resonant and FWM-nonresonant parts of the time-domain nonlinear susceptibility by representing ${\chi }^{(3)}({t}_{1},{t}_{2},{t}_{3})$ as

20 $${\chi }^{(3)}({t}_{1},{t}_{2},{t}_{3})={\chi }_{R}^{(3)}({t}_{1},{t}_{2},{t}_{3})+{\chi }_{nr}^{(3)}({t}_{1},{t}_{2},{t}_{3}),$$

with

21 $${\chi }_{R}^{(3)}({t}_{1},{t}_{2},{t}_{3})={\chi }_{R}({t}_{1})\delta ({t}_{2}-{t}_{1})\delta ({t}_{3})$$

and

22 $${\chi }_{nr}^{(3)}({t}_{1},{t}_{2},{t}_{3})={\chi }_{nr}\delta ({t}_{1})\delta ({t}_{2})\delta ({t}_{3}).$$

When transformed to the time domain and expressed in terms of ${\chi }^{(3)}({t}_{1},{t}_{2},{t}_{3})$ using Eqs (19–22), the expectation value for the anti-Stokes photon-number operator becomes

23 $$\langle {n}_{a}({\tau }_{d})\rangle \propto {\int }_{0}^{\infty }{A}_{r}^{2}(\theta -{\tau }_{d}){|{q}_{R}(\theta )+{q}_{nr}(\theta )|}^{2}d\theta ,$$

where

24 $${q}_{R}(\theta )={\int }_{0}^{\infty }d{t}_{1}{\chi }_{r}({t}_{1}){A}_{p}(\theta -{t}_{1}){A}_{s}^{\ast }(\theta -{t}_{1})$$

and

25 $${q}_{nr}(\theta )={\chi }_{nr}{A}_{p}(\theta ){A}_{s}^{\ast }(\theta ).$$

As can be seen from Eqs (23–25), the anti-Stokes signal is generally a mixture of the Raman memory readout, described by the *q*_R_(*θ*) term in Eq. (23), and FWM photons not related to the Raman memory and represented by the *q*_nr_(*θ*) term in Eq. (23). However, when the pulse widths of the pump, Stokes, and probe laser pulses *τ*_p_, *τ*_s_, and *τ*_r_, are short enough, such that *τ*_p_, *τ*_s_, *τ*_r_ ≪ 1/Γ, and the delay time *τ*_d_ is chosen such that *τ*_d_ > *τ*_p_, *τ*_s_, the FWM background in anti-Stokes counts is completely suppressed. Indeed, with ${A}_{r}^{2}(\xi )={A}_{r}^{2}\delta (\xi )$, Eq. (23) yields

26 $$\langle {n}_{a}({\tau }_{d})\rangle \propto {A}_{r}^{2}{|{q}_{R}({\tau }_{d})+{q}_{nr}({\tau }_{d})|}^{2}.$$

When *τ*_d_ > *τ*_p_, *τ*_s_, Eq. (26) reduces to

27 $$\langle {n}_{a}({\tau }_{d})\rangle \propto {A}_{r}^{2}{|{q}_{R}({\tau }_{d})|}^{2},$$

showing that, similar to time-resolved CARS with classical laser fields^29,41,42^, the expectation value for the anti-Stokes photon-number operator <*n*_*a*_> measured as a function of the delay time *τ*_d_ in the quantum version of coherent Raman scattering provides a background-free map of the |*q*_*R*_(*τ*_*d*_)|^2^ trace of Raman coherence.

Remarkably, even in this mode of quantum memory, the ratio $({{\rm{\Delta }}}_{p}^{2}+{{\rm{\Delta }}}_{s}^{2})/{{\rm{\Gamma }}}^{2}$ is meaningful as a fundamental limit on the efficiency of Raman coherence excitation as a part of memory write-in process. Indeed, the integral

28 $${\rm{\Phi }}=\int d{\rm{\Omega }}^{\prime} {\gamma }^{\ast }({\rm{\Omega }}^{\prime} )\int d{\rm{\Omega }}\gamma ({\rm{\Omega }})\int d{\omega }_{s}{A}_{p}^{\ast }({\omega }_{s}+{\rm{\Omega }}^{\prime} ){A}_{p}({\omega }_{s}+{\rm{\Omega }})\langle {n}_{s0}({\omega }_{s})\rangle $$

quantifies the amplitude of the Raman coherence excited by the pump and Stokes pulses. In the case of narrowband pump and Stokes fields, when Eq. (16) is satisfied, Eqs (12 and 28) give

29 $${\rm{\Phi }}\propto \frac{{N}^{2}{|{\alpha }_{R}|}^{4}}{{{\rm{\Gamma }}}^{2}}{P}_{p}\langle {n}_{s0}\rangle .$$

In the opposite limiting case of broadband pump and Stokes fields, with Δ_p_, Δ_s_ ≫ Γ, Eq. (28) yields

30 $${\rm{\Phi }}\propto \frac{{N}^{2}{|{\alpha }_{R}|}^{4}}{({{\rm{\Delta }}}_{p}^{2}+{{\rm{\Delta }}}_{s}^{2})}{P}_{p}\langle {n}_{s0}\rangle .$$

As can be seen from Eqs (29 and 30), higher memory operation speeds are achieved at the expense of lower efficiency of Raman coherence excitation and, hence, lower amplitude of the readout signal. Moreover, the ratio of Raman coherence excitation efficiencies in the limiting cases of narrowband and broadband laser pulses is $({{\rm{\Delta }}}_{p}^{2}+{{\rm{\Delta }}}_{s}^{2})/{{\rm{\Gamma }}}^{2}$.

Analysis presented above shows that ultrashort laser pulses provide a powerful resource for broadband quantum memories and, more generally, quantum information processing. In the Raman memory scheme considered here, the pump and probe (write and read) pulse widths are bounded from below by the oscillation period of the Raman-active phonon mode, *T*_R_ = 1/(*c*Ω_R_). For the zone-center Γ^(25+)^ (*F*_2*g*_) symmetry optical phonon in diamond, Ω_R_ ≈ 1332-cm^−1^, this limitation dictates *τ*_p_, *τ*_s_, > *T*_R_ ≈ 25 fs. Pulses shorter than 25 fs are capable of inducing a Raman excitation of the 1332-cm phonon in diamond even in the absence of a quantum Stokes (signal) field.

## Quantum memory corruption by noise four-wave mixing

Quantum memories based on Raman scattering^4,7–9,14,18^ and electromagnetically induced transparency (EIT)^3,5,21,22^ are exposed to corruption via unwanted, noise FWM pathways^43–46^. In the memory setting considered here, information stored by the Raman-active phonon may become corrupted at the write-in stage as the pump field undergoes scattering off the Raman coherence, giving rise to anti-Stokes noise photons at the frequency *ω*_a_ = *ω*_p_ + Ω_R_. In a similar FWM process, the pump field interacting with the Raman coherence at the read-out stage generates noise Stokes photons.

To understand FWM-noise-induced memory corruption in our broadband Raman memory setting, we represent the phonon coherence as a sum of the memory $(\hat{Q})$ and FWM noise $(\hat{Q})$ terms. The memory part of the Raman coherence is induced in our setting by a classical pump and a quantum Stokes field (Fig. 1a). The buildup of this coherence is governed by the quantum evolution equation (see, e.g., refs^7,30^) as dictated by the Hamitonian (4)

31 $$\frac{d\hat{Q}(\eta ,z)}{d\eta }=ig{\hat{A}}_{s}^{\dagger }(\eta ,z),$$

where *η* is the time in the retarded frame of reference.

The FWM noise that corrupts the memory is generated in our scheme by the pump that gets scattered off the Raman coherence, giving rise to an anti-Stokes photon. This part of Raman coherence builds up in accordance with the quantum evolution equation

32 $$\frac{d{\hat{Q}}_{1}(\eta ,z)}{d\eta }=ig{\hat{A}}_{a}(\eta ,z),$$

solved jointly with the equation for the anti-Stokes field generated as a result of this noise FWM,

33 $$\frac{d{\hat{A}}_{a}(\eta ,z)}{dz}=ig\hat{Q}(\eta ,z)\exp ({\rm{\Delta }}{\beta }_{a}z),$$

where Δ*β*_a_ = *β*_a_ + *β*_s_ − 2*β*_p_.

It is straightforward to see from Eq. (33) that, as long as the length of the Raman-active medium, *L*, is chosen in such a way that |Δ*β*_a_|*L* ≫ 1, anti-Stokes generation is strongly suppressed. Since the amplitude of the FWM-noise-induced anti-Stokes field controls, via Eq. (32), the buildup of the noise part of Raman coherence, large |Δ*β*_a_| can radically reduce memory corruption by FWM noise.

We now expand *β*_a_ and *β*_s_ as Taylor series about *ω*_p_:

34 $${\beta }_{a}\approx \beta ({\omega }_{p})+\frac{{{\rm{\Omega }}}_{R}}{{u}_{p}}+\frac{{\beta }_{2}}{2}{{\rm{\Omega }}}_{R}^{2},$$

35 $${\beta }_{s}\approx \beta ({\omega }_{p})-\frac{{{\rm{\Omega }}}_{R}}{{u}_{p}}+\frac{{\beta }_{2}}{2}{{\rm{\Omega }}}_{R}^{2},$$

where ${u}_{p}={(\partial \beta /\partial \omega )}_{{\omega }_{p}}^{-1}$ is the group velocity of the pump pulse and ${\beta }_{2}={({\partial }^{2}\beta /\partial {\omega }^{2})}_{{\omega }_{p}}$ is the group-velocity dispersion (GVD) parameters at the pump frequency.

Using the power-series expansions of Eqs (34 and 25), we find

36 $${\rm{\Delta }}{\beta }_{a}\approx {\beta }_{2}{{\rm{\Omega }}}_{R}^{2}.$$

It is now instructive to compare Raman memories based on Λ schemes in alkali-metal vapors and large-Ω_R_ phonons in solids with regard to the significance of phase-mismatch effects, as described by Eqs (33 and 36). For *F* = 1, *m*_*F*_ = 0 to *F* = 2, *m*_*F*_ = 0 transitions between the hyperfine-structure levels of ^87^Rb atoms, Ω_R,Rb_ ≈ 6.8 GHz. For the 1332-cm^−1^ zone-center Γ^(25+)^ (*F*_2*g*_) symmetry optical phonon in diamond, on the other hand, Ω_R,d_ ≈ 40 THz. Since |Δ*β*_a_| scales as ${{\rm{\Omega }}}_{R}^{2}$ [Eq. (36)], we take the ratio of these Raman shifts squared to find ${{\rm{\Omega }}}_{R,d}^{2}/{{\rm{\Omega }}}_{R,Rb}^{2}$≈ 3.5·10^7^. The GVD of solids is also typically much stronger than the GVD in rarefied gas vapors, as confirmed, in a different context, by early experiments on coherent anti-Stokes Raman scattering in rare-earth-metal vapors^47,48^.

Results of this qualitative analysis agree well with earlier numerical simulations^7^ and, even more importantly, are fully consistent with the results of experiments^7^, showing that phase mismatch can strongly suppress FWM noise in high-Ω_R_ diamond Raman memories. The phase-matching-enforced suppression of FWM noise and FWM-noise-induced memory corruption can be further enhanced, as pointed out in ref.^7^, by dispersion engineering in microstructured waveguides. Photonic-band-gap-assisted dispersion control of coherent anti-Stokes Raman generation has been demonstrated in earlier experiments with periodically corrugated planar waveguides^49^. Diamond is fully compatible with these technologies^50^.

## Conclusion

To summarize, we have analyzed the tradeoff between the rate at which broadband Raman quantum memories can operate and the signal-to-noise contrast of the anti-Stokes readout from such memories. In crystalline solids, the readout contrast is strongly anisotropic, spanning many orders of magnitude for various polarization arrangements of the optical fields. Off-resonance coherent four-wave mixing has been shown to provide a source of noise photons, lowering the readout contrast in broadband Raman quantum memories. We have demonstrated that this noise cannot be suppressed by phase matching, but can be reduced by many orders of magnitude with suitable polarization arrangements and proper field-waveform tailoring.

## Data Availability

All data generated or analyzed during this study are included in this article.
